# Stability and neutralization capacity of a novel mosaic HIV-1 gp140 trimer in a guinea pig model

**DOI:** 10.1186/1742-4690-9-S2-P299

**Published:** 2012-09-13

**Authors:** JP Nkolola, JM Kovacs, B Korber, B Chen, M Seaman, D Barouch

**Affiliations:** 1 BIDMC / Ragon Institute of MGH, MIT and Harvard, Boston, MA, USA; 2 Children's Hospital Boston, Boston, MA, USA; 3 Los Alamos National Laboratory, Los Alamos, NM, USA; 4 Beth Israel Deaconess Medical Center, Boston, MA, USA

## Background

The generation of globally relevant HIV-1 immunogens mimicking native, trimeric Envelope (Env) structure, remains a major challenge for HIV-1 vaccine development. We identified a mosaic Env sequence, originally designed to optimize cellular immunologic coverage of global HIV-1 sequence diversity, which was capable of forming biochemically stable trimers. We assessed this mosaic Env gp140 trimer in guinea pig immunogenicity studies compared to our previously reported biochemically stable C97ZA012 (clade C) trimer.

## Methods

Stable Env gp140 trimer derived from a synthetic mosaic sequence was stabilized with the T4-fibritin C-terminal trimerization tag and produced in 293T cells via PEI transfection. Characterization was performed by Western blotting, size-exclusion chromatography and surface plasmon resonance (SPR). Guinea pigs were immunized three times with 100 µg of mosaic or clade C gp140 protein trimer in CpG/Emulsigen adjuvants. Antibody responses were determined by ELISA and TZM.bl neutralizing antibody assays.

## Results

Stabilized mosaic gp140 trimer exhibited a single band by Western blotting, single peak by size-exclusion chromatography both after production, a freeze-thaw cycle and 7-day incubation at 4ºC. SPR analyses revealed mosaic gp140 binding with bNAb VRC01. The trimeric mosaic gp140 immunogen elicited high-titer antibodies in guinea pigs by ELISA and high-titer, cross-clade neutralization activity against tier 1 viruses. When compared to clade C gp140 trimer, NAb responses generated by mosaic gp140 trimer were 8.2- and 3.7-fold higher against clade B viruses (SF162.LS and Bal.26, respectively) but were 10.3- and 46.7-fold lower against clade A and C viruses (DJ263.8 and MW965.26, respectively).

## Conclusion

A novel, foldon-stabilized mosaic gp140 trimer elicits high-titer binding antibodies as well as high-titer, cross-clade neutralization of tier 1 viruses. The profile of the NAbs elicited by the mosaic trimer differed from that elicited by the clade C trimer. Further exploration and refinement of this concept may contribute to the development of a globally relevant Env immunogen.

